# Effect of mobile reminders on screening yield during opportunistic screening for type 2 diabetes mellitus in a primary health care setting: A randomized trial

**DOI:** 10.1016/j.pmedr.2015.08.008

**Published:** 2015-08-13

**Authors:** Sathish Kumar, Hemant Deepak Shewade, Kavita Vasudevan, Kathamuthu Durairaju, V.S. Santhi, Bhuvaneswary Sunderamurthy, Velavane Krishnakumari, Krishna Chandra Panigrahi

**Affiliations:** a Indira Gandhi Medical College and Research Institute (IGMCRI), Puducherry, India; b International Union Against Tuberculosis and Lung Disease, New Delhi, India; c Primary Health Centre, Lawspet, Department of Health and Family Welfare, Puducherry, India

**Keywords:** NPCDCS, National Programme for Prevention and Control of Diabetes, Cardiovascular diseases and Stroke, RCT, randomized controlled trial, PHC, Primary Health Centre, OPD, Out Patient Department, RBG, random blood glucose, FBG, fasting blood glucose, PPBG, postprandial blood glucose, CI, confidence interval, NNS, number needed to screen, CTRI, Clinical Trial Registry of India, HbA1C, glycosylated hemoglobin, Randomized controlled trial, Operational research, Reminder system, Diabetes mellitus, type 2, Opportunistic screening, Loss to follow-up, Primary health care, Primary Health Centre, Outpatients, India

## Abstract

*Objective*. We wanted to study whether mobile reminders increased follow-up for definitive tests resulting in higher screening yield during opportunistic screening for diabetes. *Methods*. This was a facility-based parallel randomized controlled trial during routine outpatient department hours in a primary health care setting in Puducherry, India (2014). We offered random blood glucose testing to non-pregnant non-diabetes adults with age >30 years (667 total, 390 consented); eligible outpatients (random blood glucose ≥ 6.1 mmol/l, n = 268) were requested to follow-up for definitive tests (fasting and postprandial blood glucose). Eligible outpatients either received (intervention arm, n = 133) or did not receive mobile reminder (control arm, n = 135) to follow-up for definitive tests. We measured capillary blood glucose using a glucometer to make epidemiological diagnosis of diabetes. The trial was registered with Clinical Trial Registry of India (CTRI/2014/10/005138). *Results*. 85.7% of outpatients in intervention arm returned for definitive test when compared to 53.3% in control arm [Relative Risk = 1.61, (0.95 Confidence Interval — 1.35, 1.91)]. Screening yield in intervention and control arm was 18.6% and 10.2% respectively. Etiologic fraction was 45.2% and number needed to screen was 11.9. *Conclusion*. In countries like India, which is emerging as the diabetes capital of the world, considering the wide prevalent use of mobile phones, and real life resource limited settings in which this study was carried out, mobile reminders during opportunistic screening in primary health care setting improve screening yield of diabetes.

## Highlights

• First RCT to determine the effect of mobile reminders on screening yield for diabetes mellitus.
• Operational research conducted in real world primary health care setting.
• Outpatients with random blood glucose ≥ 6.1 mmol/l among adults >30y were eligible for definitive tests.
• Mobile reminders for eligible outpatients was introduced as a reminder system.

## Introduction

In developed and developing countries, approximately 50–70% of diabetes mellitus remains undiagnosed (Mohan et al., 2006, Ramachandran et al., 2004). Opportunistic screening among adults may be a cost saving alternative or adjunct to population screening (Chatterjee et al., 2010, Evans et al., 2008, Li et al., 2010, Pereira Gray et al., 2012). It has been found that cost-effectiveness further increases by risk assessment before glucose testing (Kahn et al., 2010). Therefore under screening programs, definitive tests for diabetes are applied after an initial screening test.

Few studies from the west and India have documented the feasibility of opportunistic screening for diabetes. Available evidence suggests that after initial screening test, there was high loss to follow-up for definitive tests, resulting in low screening yield (Ealovega et al., 2004, Ginde et al., 2008, Klein Woolthuis, 2009, Shewade et al., 2015). Studies focusing on interventions to improve follow-up for definitive tests are required especially from real world primary care settings in developing countries.

India is fast emerging as the diabetes capital of the world (Mohan et al., 2007). To contain this, National Programme for Prevention and Control of Diabetes, Cardiovascular diseases and Stroke (NPCDCS) suggested opportunistic screening of persons above 30 years for diabetes mellitus (Operational guidelines. National programme for prevention and control of cancer, diabetes, cardiovascular disease and stroke (NPCDCS). Directorate General of Health Services, Ministry of Health and Family Welfare. Government of India, 2011). India is also the second largest mobile phone user in the world with 900 million users. This potential of mHealth can be used to reach out to people (Press Information Bureau, Government of India, 2012).

Hence, this study was planned to determine the effect of mobile reminders on follow-up for definitive tests and screening yield in a primary care setting in India offering opportunistic screening for diabetes to outpatients. Specific objectives were: among outpatients eligible for definitive tests for diabetes mellitus in Primary Health Centre (Lawspet), Puducherry (India) i) to compare the follow-up rates for definitive tests among those who received mobile reminders with those who did not receive mobile reminders, ii) among those with mobile reminders, to determine the screening yield, screening yield attributable to mobile reminders and etiologic fraction and iii) to determine the number needed to screen to identify one patient with diabetes mellitus.

## Material & methods

### Study design

This study was a facility-based parallel randomized controlled trial (RCT), with 1:1 allocation ratio.

### Study setting

The study was conducted in Primary Health Centre (PHC) Lawspet in Puducherry district (South India): one of the four districts in the Union Territory of Puducherry. PHC Lawspet caters to an urban population of 78,000 and has a daily patient load of approximately 150 patients in its routine Out Patient Department (OPD) and special clinics.

Routinely, opportunistic screening for diabetes at the PHC included an initial screening test, random blood glucose (RBG) for adults > 30 years, followed by definitive tests, fasting blood glucose (FBG) and postprandial blood glucose (PPBG). At PHC level, blood glucose testing was done using a glucometer. Patients from PHC were referred to district hospital for confirmation of diagnosis (clinical diagnosis using standard venous plasma glucose testing) and treatment initiation. Patients were referred back to PHC for treatment continuation.

### Study participants

All outpatients (> 30 years) attending routine OPD were included in the study. Known patient with diabetes mellitus; pregnancy; alcoholics attending OPD who were not in the state of mind to give consent; and those requiring emergency care were excluded from the study. It is to be noted that access to mobile phone even if not personally owned was not an eligibility criterion. We had planned the study with background knowledge that most of the people in our study area had access to mobile phone. Outpatients, fitting the selection criteria and willing to participate in the study (after written informed consent) were the study participants. Study participants having RBG ≥ 6.1 mmol/l were the eligible outpatients (Somannavar et al., 2009).

### Intervention and control arm

Eligible outpatients either received (*intervention arm*) or did not receive mobile reminder (*control arm*) for returning to PHC for definitive tests. Intervention was at individual level.

### Study duration

Recruitment was done between 9–18 June 2014 over 8 PHC OPD days and all eligible outpatients were followed up for 3 working days to observe for return to PHC for definitive tests. Recruitment was stopped once desired sample size was reached.

### Sample size and randomization

Sample size calculation was done for hypothesis testing for two proportions (large proportion — equal allocation) using nMaster sample size calculator 1.0 software developed by Christian Medical College, Vellore, India. Assuming proportion of eligible outpatients returning for definitive tests in intervention arm and control arm (*primary outcome*) to be 60% and 30% (Shewade et al., 2015) respectively; an alpha error of 5%; and power of 95% a minimum of 64 eligible outpatients were required in each arm. To allow for one sub-group analysis we doubled the sample size in each arm (n = 128).

Central randomization was used to randomize eligible outpatients into intervention and control arm. Computer generated random allocation sequence (block randomization, block sizes of four and six; random selection of blocks) was prepared beforehand and available with a statistician who didn't belong to the investigation team.

### Procedure

The investigator was present next to the OPD registration counter. As soon as an outpatient was registered in the OPD and given an OPD slip, s/he was given the option of recruitment into the study by the investigator. After written informed consent, study participants were subjected to RBG testing by the investigator using a glucometer. All eligible outpatients were provided an investigation slip and asked to follow up for definitive test (in fasting state) on the next working day. After this, the investigator guided the patient to the medical officer chamber for OPD consultation. A mark was made on the top of the OPD slip of all eligible outpatients which hinted the medical officer to reinforce follow-up visit for definitive tests. After consultation, the medical officer also requested all eligible outpatients to meet the laboratory technician. The laboratory technician in addition to reinforcing the follow-up visit, in detail described what fasting state meant. The laboratory technician maintained (outcome assessor) register containing FBG and PPBG value of eligible outpatients who followed up. Each study participant was given a unique identifier which was used to trace the patient from initial screening test to definitive test.

After OPD on every afternoon, the investigator prepared a list of eligible outpatients in Microsoft Excel, with information on unique identifier. The excel sheet was emailed to the statistician who the same afternoon replied (through email) with arm allocation against each unique identifier. Those in intervention arm received a mobile reminder (a call on the same evening) by the investigator requesting them to come for definitive tests. In case they could not be reached in one call, maximum of three calls were made (each one hour apart). A call script was used uniformly for the mobile reminders.

All tests were performed on capillary blood (pin prick) using a glucometer (One Touch Select Simple Glucose Meter). Glucometers were standardized every morning against a standard glucose solution. For epidemiological diagnosis, FBG ≥ 7 mmol/l or PPBG ≥ 11.1 mmol/l was considered as diabetes mellitus. FBG between 6.1 and 6.9 mmol/l or PPBG between 7.8 and 11 mmol/l was considered as pre-diabetes (Definition and diagnosis of diabetes mellitus and intermediate hyperglycaemia: Report of a WHO/IDF consultation, 2006). Blinding of eligible outpatients was not possible for obvious reasons. Blinding was done at the level of outcome assessment and data analysis.

### Data management and analysis

Data collected was recorded in a data collection form. Variables collected from study participants included: unique identifier (serial number), date of OPD, age, sex, RBG, eligible outpatient (yes/no), study arm(intervention/control/not applicable), call attended (yes/no/not applicable), follow-up done (yes/no/not applicable)), FBG and PPBG. Data were double entered, validated and analyzed using EpiData (version 3.1 for entry and version 2.2.2.182 for analysis, EpiData Association, Odense, Denmark).

Continuous variables were summarized using mean and standard deviation. Proportions, relative risks (adjusted for confounding wherever applicable using mantel Haenszel method) and 95% confidence intervals (CI) were calculated. Chi square test was used for statistical significance. Intention to treat analysis was used. Screening yield, attributable screening yield, etiological fraction and number needed to screen (NNS) were also calculated. Screening yield was calculated as the number of diabetes patients detected divided by the total number of study participants. Difference of screening yield in intervention and control group was attributable screening yield. NNS was calculated as inverse of attributable screening yield and etiological fraction was calculated by dividing attributable screening yield by screening yield in intervention group. We shall be reporting the cost-effectiveness of the intervention in a separate paper.

### Ethics considerations

The study was approved by the local Institute Ethics Committee. The study was registered with Clinical Trials Registry — India (CTRI/2014/10/005138). Patient information sheet and consent form were prepared both in English and in the local language (Tamil). Routine OPD care of the outpatients was not affected. Eligible outpatients who did not follow up for definitive tests within 3 days were traced and followed up by the PHC for further management. Study participants found to be having diabetes or pre-diabetes were provided with standard care in the PHC. Cash incentives or incentives of any other form were not given to study participants.

## Results

### Baseline characteristics

A total of 667 non-pregnant non-diabetes adult (> 30 years) patients attended OPD at PHC during the intervention period; of which 390 (58.5%) agreed to be part of the study (study participants). Baseline characteristics of the study participants were as follows: 207 (53.1%) were males, mean (SD) age was 44.9 (10.7) years and mean (SD) RBG was 7.7 (3.3) mmol/l.

Of 390 study participants, 268 (68.7%) had RBG ≥ 6.1 mmol/l (eligible outpatients). Of the eligible outpatients, 133 were randomized to intervention arm and 135 to control arm. Baseline characteristics of eligible outpatients have been summarized in Table 1. Proportion of males was higher in intervention arm when compared to control arm, while difference in distribution of age and RBG was not clinically significant. In the intervention arm, all had access to a mobile phone even if not personally owned and 120 (90.2%) attended the call (mobile reminder).

### Primary outcome

**Fig. 1:**
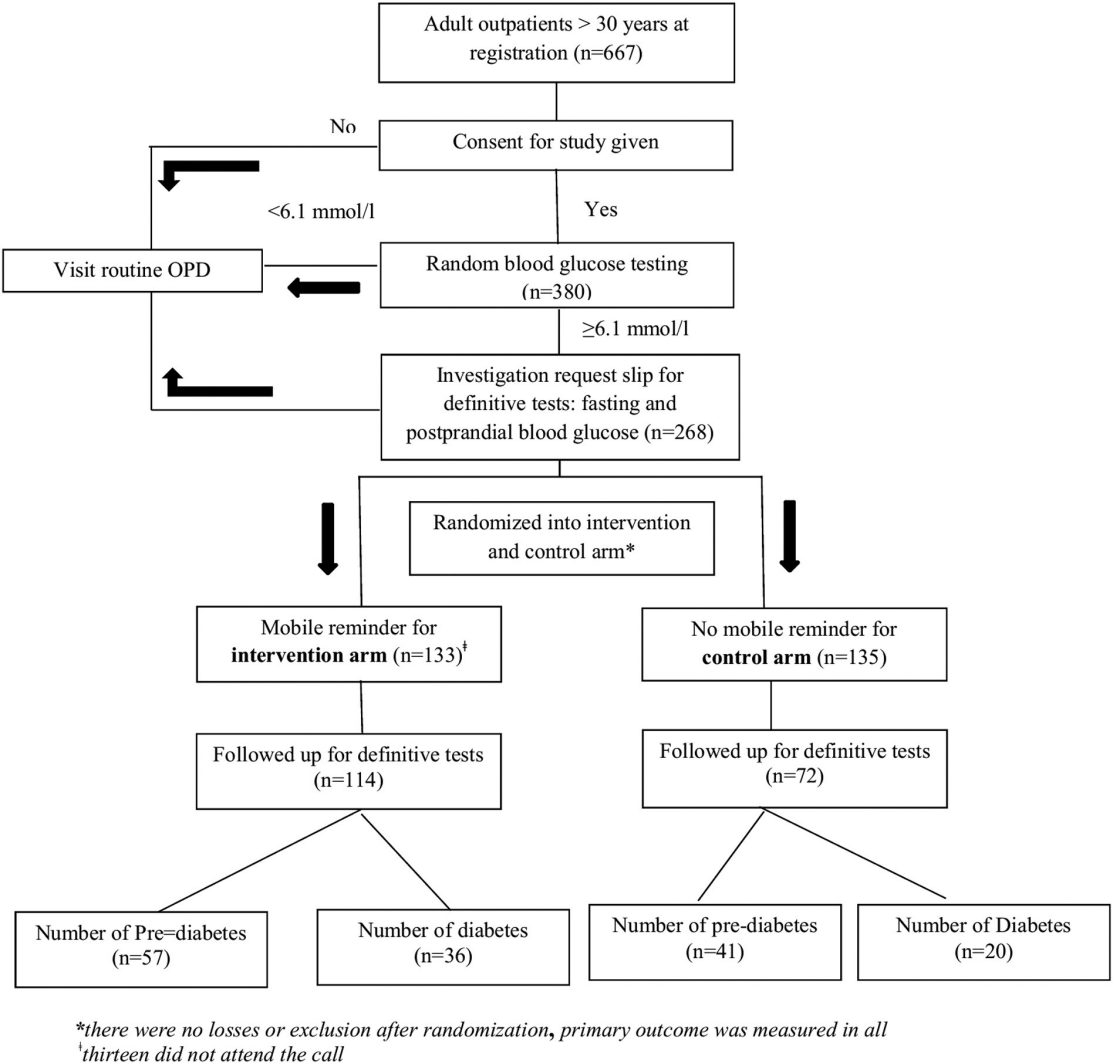
Participant flow during opportunistic screening for diabetes at Primary Health Centre, Lawspet, Puducherry, India (2014).

Our data showed that 85.7% of outpatients in intervention arm returned for definitive test when compared to 53.3% in control arm. In other words outpatients with mobile reminder had 1.6 times more chance of returning for definitive tests and this was statistically significant [RR = 1.61, (0.95 CI — 1.35,1.91), p < 001]. Relative risk adjusted for sex was 1.59 (1.34, 1.88), p < 0.001. Number of patients who were diagnosed with diabetes in intervention and control arm were 36 (27.1%) and 20 (14.8%) respectively. Number of patients who were diagnosed with pre-diabetes in intervention and control arm were 48 (36.1%) and 31 (23%) respectively. Outpatient flow has been depicted in Fig. 1.

Considering inclusion of 31.3% patients that were not eligible for definitive tests in the denominator (RBG < 6.1 mmol/l, n = 122), screening yield for diabetes in intervention and control arm was 18.6% (36/194) and 10.2% (20/196) respectively. Attributable screening yield was 8.4%. Etiologic fraction was 45.2%.and NNS was 11.9.

## Discussion

This is the first randomized trial in a real-world primary care setting of a developing country offering outpatients opportunistic screening for diabetes. Eligible participants (age > 30 years and RBG ≥ 6.1 mmol/l as initial screening test) who received mobile reminders showed significant improvement in follow-up for definitive tests, compared to control participants with no reminders.

Studies in the past have evaluated the effect of various reminder systems on screening rates for diabetes. Screening rate in these studies had been defined as “percentage of adults covered”. A RCT from New Zealand showed that patient and/or computer reminders resulted in increased screening rates in general practice (Kenealy et al., 2005). Among mothers having gestational diabetes mellitus, a RCT had shown that postal reminder to patient, physician or both was associated with 3 times increase in screening rate (oral glucose tolerance test within a year of delivery) (Clark et al., 2009). Similarly, letter or phone reminder in Canada resulted in 2 times increase in screening rates within 6 months after delivery (Shea et al., 2011) and a retrospective review from Canada also showed 3 fold increase in odds of being screened due to a reminder checklist (Lega et al., 2012).

Once covered under a screening program, studies have documented low screening yields worldwide citing high loss to follow-up as the main reason (Ealovega et al., 2004, Ginde et al., 2008, Klein Woolthuis, 2009, Shewade et al., 2015). There were no studies found during literature review that determined the effectiveness of a reminder system on improvement in follow-up for definitive tests and screening yield. Our study did not deal with improvement in screening rates through mHealth but with improvement of screening yield among those screened.

Mobile reminder performed by the investigator can be done by the laboratory technician in real world settings, simple honorarium or allowance may be given to the person concerned. An automated telephone conversion may be an alternative to mobile reminder by the laboratory technician. It has been suggested that automated telephone conversion has a role to deliver behavioral interventions and may be interfaced into the general health care delivery system (Friedman, 1998).

Installation of a centrifuge for plasma separation at PHC level may be done for venous plasma testing. Alternatively, definitive tests used in this study can be replaced with HbA1C testing depending on the availability of a standard laboratory, with a caution that HbA1C detects more cases of diabetes (Nazir et al., 2012). HbA1C also has the additional advantage of not requiring a fasting sample; blood can be collected the same day thus improving follow-up. Hand-held point of care testing using HbA1C is another option (Marley et al., 2015). Outsourcing of tests at PHC level for venous plasma glucose testing or HbA1C may be done.

### Policy implications

This study has provided some useful information which would be of interest to policy makers as well. *First*, 45% of screening yield in intervention arm was attributable to mobile reminder. In other words, during routine opportunistic screening if we identify 10 cases with diabetes among 100 screened; then introduction of mobile reminders would identify 8 additional cases with diabetes. Eleven persons will have to be screened using RBG, followed by mobile reminder for follow-up (if eligible) and administration of definitive tests, to diagnose one case with diabetes mellitus. Considering this, the intervention appears to be cost-effective on face value; however, a systematic economic analysis needs to be carried out before coming to this conclusion. *Second*, alternate modes of mobile reminder delivery may be tried. *Third*, it seems odd that clinical diagnosis is neither made nor expected to be made at PHC level (NPCDCS operational guidelines). There could be a case for clinical diagnosis at PHC and training medical officers to diagnose and manage uncomplicated diabetes at PHC level and use referral services to district hospitals only for target organ damage investigations and complicated diabetes.

### Strengths and limitations

Our intervention was conducted as an operational research with existing manpower and other resources in the PHC. This model was operationally simple, conducted in real world setting without affecting the routine functioning of OPD, therefore, feasible and replicable in other resource poor settings. Patients generally find screening process lengthy and unacceptable (Eborall et al., 2012). In such a scenario, we had decided against collecting information on other possible confounders as this would have slowed down the OPD. We expect randomization to have taken care of them. Timing of randomization was apt and therefore, request for follow-up for definitive tests was truly blinded. Follow-up period was common for all eligible patients irrespective of study arms. Data were quality assured and robust as double data entry and validation was done. CONSORT guidelines were adhered to while reporting the findings of this RCT (Schulz et al., 2010).

Short follow-up period for return for definitive tests was a major limitation of our study. However, assuming that patients who do not return within three working days have a very less chance of returning later and any change in follow-up would have been similar in both the arms, it would be safe to presume that increasing the follow-up period might not have impacted the results significantly. We do not rule out the fact that baseline health seeking behavior and informed consent itself might have prejudiced follow-up for definitive tests (increased); however, we expect this to be same in both the arms.

RBG testing has its own limitations. However, by reducing the cut off to 6.1 mmol/l false positives and false negatives were reduced to a large extent. In fasting state, capillary blood glucose is equal to venous blood glucose. In non-fasting state, capillary blood glucose is higher than venous blood glucose (Somannavar et al., 2009). Also, plasma glucose is higher than blood glucose (Kempe et al., 1997, Kuwa et al., 2001). For the above reasons capillary blood glucose cannot be a replacement for venous plasma glucose which is required for clinical diagnosis. PHC didn't have facility for plasma glucose testing and repeat measurement was not done. Therefore, the diagnosis of diabetes in our study was epidemiological using WHO guidelines (Definition and diagnosis of diabetes mellitus and intermediate hyperglycaemia: Report of a WHO/IDF consultation, 2006).

## Conclusions

In countries like India, which is emerging as the diabetes capital of the world, considering the wide prevalent use of mobile phones, and real life resource limited settings in which this study was carried out, mobile reminders during opportunistic screening in primary health care setting improve screening yield of diabetes. The intervention appears effective and feasible for successful replication in the region. *Future studies* should focus on translational research on how best to implement mobile reminders. Also, in settings where venous plasma testing is not possible at PHC level, studies are required to systematically study loss to follow up of patients referred to district hospital for clinical diagnosis.

## Funding statement

This was an operational research carried out using available resources and manpower at the Primary Health Centre. The authors declare that there was no source of funding separately for the research.

## Contributor statement

Conception and design of the protocol (SK HDS KV KCP), data capture tool development (HDS), acquisition of data (SK KS VSS KKV), data analysis/interpretation (SK HDS BS), drafting and critically reviewing the paper (all), approval of final version (all).

HDS is the guarantor of this work and, as such, had full access to all the data in the study and takes responsibility for the integrity of the data and the accuracy of the data analysis.

## Conflict of interest statement

The authors declare that there are no conflicts of interest.

## Figures and Tables

**Table 1:** Baseline characteristics of eligible outpatients^a^ during opportunistic screening for diabetes at Primary Health Centre, Lawspet, Puducherry, India (2014) (N = 268).

Variable	Intervention arm (n = 133)	Control arm (n = 135)
Sex [n (%)]		
• Male	74 (55.6)	61 (45.2)
• Female	59 (44.4)	74 (54.8)
Age in years [mean (SD)]	46.5 (11.2)	44.6 (11)
RBG^b^ mmol/l [mean (SD)]	8.7 (3.7)	8.7 (3.4)

% column percentage.

a Adult outpatients > 30 years & random blood glucose ≥ 6.1 mmol/l.

b Random blood glucose.
